# Editorial

**Published:** 1893-12

**Authors:** 


### HOMŒOPATHIC PHYSICIAN,

A MONTHLY JOURNAL OF

HOMŒOPATHIC MATERIA MEDICA AND CLINICAL MEDICINE.

"If our school ever gives up the strict inductive method of Hahnemann, we are lost, and deserve only to be mentioned as a caricature in the history of medicine."—CONSTANTINE HERING.

Vol. XIII. DECEMBER, 1893. No. 12.

### EDITORIAL.

ADDRESS TO DELINQUENT SUBSCRIBERS.—With this number we close our account with the year 1893. Not so with many of our subscribers. Their accounts with this journal are not closed. On looking over our subscription-list we find a discouragingly large number who are still in debt for the journal. No doubt many of these readers think that the amount which he or she owes is so trifling that it makes no difference whether it is paid or not. So many think this way that the aggregate sum which they all withhold amounts to a very large figure—several thousands of dollars.

Inability to collect the individual fractions which make this sum puts the publisher to serious inconvenience in issuing the journal each month. Printing bills must be paid, and the editor cannot pay them out of his own pocket. The subscribers ought to support the journal loyally, willingly, without the necessity of cumbering the editorial pages with this appeal. We think that the material which we give the profession from month to month is of such a high order of merit that it should inspire our readers with a cordial desire to show their appreciation of the work done for their benefit by spontaneously making a free-will offering for its support.

The material given is such as is useful to them in everyday practice. It is designed to be a practical assistant and guide. Or it is calculated to awaken their interest in, understanding of, and courage to practice a more efficient and successful mode of treatment than any heretofore known in any age of the world. It is only of late years that the several so-called homœopathic journals have made any efforts whatever to instruct their readers in the system of Hahnemann.

*This* journal has always kept this idea steadily in view. The completed volumes are valuable works of reference. Our readers tacitly admit this assertion by the careful way in which they save their numbers and have them bound into volumes. A surprisingly large number do this, and keep the volumes on their shelves. Even when a stray set of volumes does get into the hands of a second-hand dealer it is not held by him very long. He quickly finds a purchaser.

Such being the case, it follows that THE HOMŒOPATHIC PHYSICIAN is well worthy to be maintained by the profession.

We now ask them to indorse this sentiment by remitting the sums due us without further delay, so that funds may be ready to meet the demands of a new year.

Failure to collect enough to meet the expenses will compel the suspension of publication; and there is a very large circle of friends of the journal who, we know, will not like that event.

---

WELLS ON INTERMITTENT FEVER.—A few pages yet remain to be printed of this valuable publication. It was not possible to put them into type for the November and December numbers because of the scarcity of funds to meet the printing bills. It is very difficult work for the printer, and therefore is very expensive. Indeed, few compositors can set up such work at all. The present printers who set the type for these pages have had ten years' training and experience upon this journal alone, and are therefore more than ordinarily competent. But then *they must be paid*. Being expert upon such a difficult task, they are more than ordinarily worthy, and, knowing their worth, expect prompt pay for their services. Their demands can be met only by our subscribers "chipping in" with their long overdue remittances. Will they help us in our good work?

